# Trends in Informal Payments by Patients in Europe: A Public Health Policy Approach

**DOI:** 10.3389/fpubh.2021.780337

**Published:** 2021-11-22

**Authors:** Adrian V. Horodnic

**Affiliations:** Faculty of Medicine, “Grigore T. Popa” University of Medicine and Pharmacy, Iaşi, Romania

**Keywords:** informal payments by patients, institutional misalignment, institutional theory, norms, values, social norms

## Abstract

**Background:** A new institutional approach toward informal payments in healthcare views informal payments as arising when there is a misalignment between values/norms (informal institutions) and the formal rules (formal institutions) of patients. However, less knowledge is available on the effectiveness of this approach in tackling informal payments in healthcare. This study aimed to fill this gap by evaluating the trends in the effect of institutional misalignment on informal payments made by patients.

**Methods:** A quantitative study design with data extracted from the last three waves of special Eurobarometer surveys on corruption was used to model the propensity of European patients in 27 European Union countries and the United Kingdom to make informal payments. Multilevel logistic regression analysis was employed in order to test the relationship between the formal–informal institution misalignment and the likelihood to make informal payments. Sensitivity analyses were also performed to test the robustness of the findings.

**Results:** The finding is that there is a strong association between the formal–informal institution misalignment and the likelihood to make informal payments for public healthcare services. Similarly, social norms play a pivotal role. When patients perceive that informal practices are widespread in the public healthcare sector they are more likely to make informal payments themselves.

**Conclusion:** The outcome is a call for complementing deterrence measures toward informal payments in healthcare with measures aiming to reduce the formal–informal institution misalignment and to change the social norms. This can be achieved by improving the structural conditions at country level and by changing values/norms and beliefs of patients.

## Introduction

In the past decades, a growing literature reveals how patients make direct contributions in cash or in-kind for healthcare services to which they are entitled to. These contributions are additional to the required official contribution (1). Also defined as “unofficial payments,” “under-the-table payments,” or “under-the-counter payments” (2–4), the phenomenon is not a minority practice, affecting both developed and developing countries (5–10). However, the finding of the previous studies is that there is a high variance in the prevalence of patients who make informal payments, with a higher prevalence in the post-socialist countries [i.e., from 35 to 60% of patients accessing healthcare services in Bulgaria, Lithuania, Poland, Hungary, Romania, and Ukraine (11)]. The widespread practice of informal payments in healthcare is inefficient, and results in many patients being unable to pay for the care they need (7, 11–13). Tackling informal payments are essential for providing to the patients more equal access to the care they need and for building up a health system which does not rely on corruption and bribes. The phenomenon is, therefore, a core issue of public health and represents a top priority for both governments and supranational agencies (14).

When explaining informal payments by patients, previous literature found focused on determinants related mainly to economic conditions (e.g., lack of funding) or governance failures (e.g., lack of transparency, poor accountability) (15–18). Indeed, it has been revealed that these informal payments are made to obtain better treatment (19, 20), a supplementary service (4), to express gratitude (19, 21) because the “doctor demanded payment” (19) or due to their concern of being refused the treatment (22). Healthcare professionals usually accept informal payments due to their low salaries (23). As such, only a small number of studies aimed at explaining informal payments more deeply, beyond finding a range of motives and/or systemic determinants (15, 16, 21), and even fewer used some broader theorisations of this practice (i.e., the importance of values, norms, and beliefs) (24). One valuable theorisation of this practice that accounts for the role of the patients' values, norms, and beliefs is represented by the institutional asymmetry thesis, a new-cutting edge theorisation developed in 2017 (25, 26). Employing institutional theory framework, informal payments are considered to arise because the values, norms, and beliefs of the informal institutions (“civic morale”) are in misalignment with the formal rules (codified laws and regulations of the formal institutions of society—“state morale”). As such, this explanation captures the asymmetry between the patients' individual values/norms and the formal regulations in the public healthcare sector. Drawing from this theorisation, the following hypothesis is proposed to be tested:

H1: The higher the formal–informal institution misalignment the greater the propensity to make informal payments to healthcare practitioners in the public healthcare sector.

Surprisingly, despite a large number of studies investigating the informal economy which underline the important role of the social norms (27–31), this has been under-researched in respect of the informal payments made by patients (24). Social norms represent the trust between the actors in a society (32) and, in this context, amongst the patients and healthcare professionals. Thus, according to this view, the probability that healthcare professionals and patients accept/make informal payments is higher if they live in a community where this practice is deemed to be widespread and to represent a social norm. This is because they would worry less about the potential sanctions and also, they perceive that “everybody does it” (19). Therefore, the following hypothesis is proposed here to be tested:

H2: The higher the perceived widespread of informal practices in the public healthcare sector the greater the propensity to make informal payments to healthcare practitioners in the public healthcare sector.

In summary, up to date, there is less understanding on informal payments made by patients, and neither previous study sought to investigate the trends over time in the prevalence of this phenomenon nor to evaluate the trends in the effect of values, norms, and beliefs on the informal payments made by patients. This study aimed to fill this gap by reporting the results of three Eurobarometer surveys on the corruption in the European Union Member States and the United Kingdom (UK). As such, theoretically, by testing the proposed hypotheses, this paper reflects and develops a new cutting-edge theorisations toward the informal payments made by patients for further advancing the new institutional asymmetry thesis toward the informal payments by patients promulgated in 2017 (25, 26) by integrating the issue of social norms (i.e., expectations of patients and healthcare professionals about other peer citizens to behave in uncorrupted manner). Methodologically, the major contribution of this paper is the regression analysis conducted across three different waves of the Eurobarometer survey in order to test the robustness of the findings over the years. Empirically, this paper contributes to explaining and tackling this phenomenon. The next section describes in detail the employed data and methods. This is followed by the results section including both, descriptive statistics and multivariate analysis and finally, the conclusion section discusses the implication of the findings.

## Methods

### Data

This paper uses the results of three Eurobarometer surveys on the corruption in the European Union Member States and the UK, namely, Special Eurobarometer No. 397 conducted in 2013 (27,786 respondents), Special Eurobarometer No. 470 conducted in 2017 (28,080 respondents), and Special Eurobarometer 502 conducted in 2019 (27,731 respondents). These surveys involved face-to-face interviews with adults aged over 15 years at the home of respondents. The sample design involved a multi-stage random (probability) approach and ensured appropriate coverage according to the population density in each country included in the sample. In order to ensure that the obtained sample reflects the universe structure, a national weighting procedure was employed based on gender, age, region, and locality size (33–35). The resultant sample ranges from ~500 respondents in smaller Member States to more than 1,500 respondents in larger countries. Those respondents who had been to a healthcare practitioner in a public healthcare facility in the past 12 months (before the survey took place) were used for analytical purposes, resulting in a sample of 21,121 patients for the survey conducted in 2013 and 21,623 and 20,763 patients for the surveys conducted in 2017 and 2019, respectively.

Although the face-to-face interviews in the three Eurobarometer waves covered various issues involving corruption practices, the discussion is here confined to questions related to the use of informal payments paid to healthcare practitioners in public healthcare institutions. This is based on a dummy variable with a coded value 1 if patients had to make informal payments and 0 otherwise. To evaluate whether there is an association among the values, norms, and beliefs of patient and the probability to make informal payments, a composite index measuring the misalignment between these norms (informal institutions) and the formal rules (formal institutions) is firstly constructed. Each patient rated the perceived acceptability of three examples of corrupt behaviors (to give gifts, money, and to do favors to get a “free” public service) on a scale from 1 (always acceptable) to 3 (never acceptable). Similar to other studies (25, 36, 37), these three attitudinal questions were used to compute the asymmetry index, a lower value indicating that informal institutions (values, norms, and beliefs of a patient) are in asymmetry with formal institutions (formal rules). Secondly, the perception on whether the abuse of power for personal gain as well as giving/taking bribes are practices that are widespread in the healthcare system is here evaluated (social norms). Based on this, a dummy variable is computed, with value 1 if patients consider that these informal practices are widespread in the healthcare system and 0 otherwise. In addition, akin to other studies on informality (38–40), fraud in healthcare (41) or informal payments (42–45), a range of individual-level socio-demographic variables are here used as control variables (gender, education, household type, financial status, and the size of the community; details in Supplementary Table 1 in the Supplementary Material).

### Analytical Methods

In order to explore the relationship between patients' values, norms, and beliefs and the propensity to make informal payments over time, descriptive analysis on the variation of this practice across countries and multi-level logistic regression analysis are employed. Indeed, considering the hierarchical structure of the data (patients within countries), we tested whether a multi-level logistic regression analysis should be used. The tests showed that 28% of the variance in the likelihood to make informal payments is due to country-level characteristics. The null model ran with 2013 data from the Special Eurobarometer No. 397 showed statistically significant disparities between the evaluated countries in the prevalence of informal payments (Wald test = 12.01, *p* < 0.001). Similar results are obtained when running the null models with 2017 and 2019 data from the Special Eurobarometers No. 470 and No. 502 (Wald test = 11.79, *p* < 0.001; Wald test = 10.79, *p* < 0.005, respectively), revealing that multi-level logistic regression should be the one used. For the descriptive statistics, the sampling weighting scheme was used as recommended in the methodology of the Special Eurobarometer (33–35). However, for the multivariate analysis, various estimations were computed considering the debate in the literature overusing the weighting scheme (46, 47). Marginal effects of patients' values, norms, and beliefs and of socio-demographic variables as well as predicted probabilities to make informal payments are graphically displayed to provide a visual view of the magnitude of their impact on the likelihood to make informal payments over time. Moreover, a robustness analysis is conducted to further test the results obtained. Firstly, the multi-level logistic regression analysis is conducted (a) with weighting scheme and (b) with imputed missing data (for details: Supplementary Table 2 in the Supplementary Material). Secondly, considering that informal payments are observable only for those who had been to a healthcare practitioner in a public healthcare institution, a Heckman selection model is also estimated to control for the selection issue, with and without weighting scheme. Based on the results and the recommendations in previous studies (48, 49), a selection equation with variables related to the use of healthcare services (age, education, and household size) was included. Third and finally, logistic regression is conducted (a) without weighting scheme (clustered by country), (b) with weighting scheme (including country dummies), and (c) with imputed missing data (for details: Supplementary Table 2 in the Supplementary Material). The next section reports the findings.

## Results

Starting to analyse the trend in the prevalence of informal payments made by patients to healthcare practitioners in a public healthcare institution, and as Table 1 shows, at the European level, there is no clear-cut path. In 2019, about 5% of the patients declared that they made informal payments to the healthcare professionals from public healthcare institutions which represent an increase comparing to 2017 (when only 4% of patients declared making such payments) and similar figures in 2013. It is important to acknowledge that out of the 28 analyzed countries, only in three countries there is a descending trend in the percentage of patients making informal payments to healthcare, namely in Romania (19% in 2019 compared to 28% in 2013), Lithuania (10% in 2019 compared to 21% in 2013) and Sweden (0% in 2019 compared to 1% in 2013). In France, Estonia, Italy, the Netherlands, and Slovenia, the percentage of patients making informal payments to healthcare professionals remained unchanged between 2013 and 2019. Meanwhile, as Table 1 displays, most of the countries followed an increase in the percentage of patients making informal payments, such as Austria, Belgium, Bulgaria, Croatia, Czech Republic, Denmark, Finland, Greece, Ireland, Latvia, Luxembourg, Malta, and the United Kingdom. For the rest of the countries, the trend is not clear-cut, observing either an initial increase in 2017 followed by a decrease in 2019 (Cyprus, Hungary, and Poland) or the other way around (Germany, Portugal, Slovakia, and Spain).

**Table 1:** Trends in informal payments to healthcare practitioners in the EU-27 and the UK (%; 2013, 2017, 2019).

**Region/Country**	**Special Euro barometer 397 (2014, fieldwork 2013)**	**Special Euro barometer 470 (2017, fieldwork 2017)**	**Special Euro barometer 502 (2020, fieldwork 2019)**	**Trend**
EU-27 and UK	5	4	5	Uncertain
Romania	28	19	19	Descending
Austria	3	9	17	Ascending
Greece	11	13	14	Ascending
Hungary	10	17	14	Uncertain
Bulgaria	8	8	10	Ascending
Latvia	7	8	10	Ascending
Lithuania	21	12	10	Descending
Luxembourg	1	5	9	Ascending
Germany	8	4	7	Uncertain
Croatia	2	3	7	Ascending
Belgium	2	5	6	Ascending
Czech Republic	4	4	5	Ascending
France	5	5	5	Unchanged
Poland	3	7	5	Uncertain
Slovakia	9	4	5	Uncertain
Italy	4	4	4	Unchanged
Malta	2	4	4	Ascending
Estonia	3	3	3	Unchanged
Ireland	2	2	3	Ascending
Slovenia	3	3	3	Unchanged
Denmark	1	2	2	Ascending
Spain	1	0	2	Uncertain
Cyprus	2	3	2	Uncertain
Portugal	2	1	2	Uncertain
United Kingdom	1	1	2	Ascending
Netherlands	1	1	1	Unchanged
Finland	0	1	1	Ascending
Sweden	1	1	0	Descending

*Don‘t know/Refusal included*.

*Source: own calculations based on data from Special Eurobarometer 397/Wave EB79.1 (2014, fieldwork 2013), Special Eurobarometer 470/Wave EB88.2 (2017, fieldwork 2017) and Special Eurobarometer 502/Wave EB92.4 (2020, fieldwork 2019)*.

In order to explore which policy measures could be more effective in reducing informal payments made by patients to public healthcare practitioners, Table 2 shows which of the individual characteristics of patients are significantly associated with the propensity of making such payments. The results of the multilevel logistic regressions show that while for the socio-demographic characteristics, the effect is not constant, being vanished in some of the survey waves, the attitudinal characteristics (values, norms, and beliefs) are strongly associated with the likelihood of making informal payments across the analyzed period (2013, 2017, and 2019). Indeed, according to the all models, the higher the asymmetry index of patients between the formal institutions and informal institutions (i.e., high misalignment), the higher the likelihood of making informal payments to healthcare practitioners in the public healthcare sector (confirming Hypothesis 1). Similarly, when patients consider that the informal practices are widespread in the healthcare system it is more likely that they, themselves, will make such informal payments (confirming Hypothesis 2).

**Table 2:** Multilevel logistic regression of the propensity to make informal payments for healthcare services in EU-27 and the UK—trends 2013, 2017, 2019.

	**Model 1**		**Model 2**		**Model 3**	
	**Special Eurobarometer 397 (2014, fieldwork 2013)**		**Special Eurobarometer 470 (2017, fieldwork 2017)**		**Special Eurobarometer 502 (2020, fieldwork 2019)**	
**Fixed part**	**Coef**.	**SE^a)^**	**Coef**.	**SE^a)^**	**Coef**.	**SE^a)^**
Values, norms, and beliefs						
Asymmetry Index^b)^	−1.233^***^	(0.072)	−1.090^***^	(0.066)	−1.309^***^	(0.061)
Widespread IPH^c)^						
Yes	0.768^***^	(0.079)	0.779^***^	(0.077)	0.464^***^	(0.071)
Socio-demographic variables						
Gender (R: Male)						
Female	0.099	(0.072)	0.001	(0.070)	−0.150^**^	(0.066)
Age education ended (R: Up to 15 years)						
16–19 years	0.056	(0.109)	0.138	(0.111)	0.185^*^	(0.109)
20+ years	0.245^**^	(0.118)	0.444^***^	(0.116)	0.332^***^	(0.115)
Still studying^d)^	−0.166	(0.184)	0.097	(0.199)	0.039	(0.186)
Household (R: Single hh^e)^ without children)						
Single hh with children	−0.141	(0.156)	0.275^*^	(0.156)	0.036	(0.155)
Multiple hh without children	0.003	(0.093)	0.464^***^	(0.094)	0.256^***^	(0.087)
Multiple hh with children	0.123	(0.091)	0.428^***^	(0.096)	0.258^***^	(0.092)
Financial difficulties^f)^ (R: Most of the time)						
From time to time	0.016	(0.099)	−0.111	(0.115)	−0.279^**^	(0.111)
Almost never/never	−0.241^**^	(0.103)	−0.369^***^	(0.115)	−0.651^***^	(0.112)
Community size (R: Rural area or village)						
Small or middle sized town	0.118	(0.086)	0.035	(0.086)	0.101	(0.085)
Large town	0.102	(0.090)	0.127	(0.089)	0.418^***^	(0.083)
Constant	−0.669^**^	(0.287)	−1.106^***^	(0.284)	0.156	(0.266)
Random part						
Country-level variance	0.710		0.680		0.610	
(Standard error)	0.215		0.208		0.196	
Variance: country level (ICC) (%)	17.75		17.13		15.64	
Observations	20,019		20,633		19,873	
Countries	28		28		28	
Wald chi2	413.82		427.39		609.76	
Prob. > chi2	0.0000		0.0000		0.0000	

*Significant at*

* *p < 0.1*,

** *p < 0.05*,

*** *p < 0.01; Coefficients compared to reference category (R)*.

a) *Standard Errors*;

b) *Values, norms, and beliefs (informal institutions) in asymmetry with formal rules (formal institutions)*;

c) *IPH, Informal Practices in the Health sector; Perception that informal practices (giving/taking bribes, abuse of power for personal gain) are widespread in the healthcare system*;

d) *No full-time education included*;

e) *Household*;

f) *In paying household bills*.

*Source: own calculations based on data from Special Eurobarometer 397/Wave EB79.1 (2014, fieldwork 2013), Special Eurobarometer 470/Wave EB88.2 (2017, fieldwork 2017) and Special Eurobarometer 502/Wave EB92.4 (2020, fieldwork 2019)*.

These results are robust when other types of regression analyses are employed, regardless, if weighting scheme, imputation of the missing values or sample selection issues are considered (details in Table 3). Moving to the socio-demographic characteristics, the results show that financial status plays an important role (consistent in all three analyzed survey waves), those who never or almost never face financial difficulties being less likely to pay informally healthcare practitioners. Similarly, consistent findings across the survey waves were found for the education level, those who ended education at 20 years old or later are more likely to informally pay healthcare practitioners. Meanwhile, for other characteristics such as gender, household size or community size, the association with the propensity of making informal payments is vanished in some of the survey‘s waves.

**Table 3:** Robustness analysis.

			**Norms, values, and beliefs**		**Other variables**		**Selection equation^d)^**	**N^e)^**	**I^f)^**	**Prob. >chi2/F**
			**Asymmetry index^a)^**	**Widespread IPH^b)^**	**SocD^c)^**	**Country**				
Multilevel logistic regression	Without weights	2013	−1.233^***^ (0.072)	0.768^***^ (0.079)	Yes			20,019		0.000
		2017	−1.090^***^ (0.066)	0.779^***^ (0.077)	Yes			20,633		0.000
		2019	−1.309^***^ (0.061)	0.464^***^ (0.071)	Yes			19,873		0.000
	With weights	2013	−1.233^***^ (0.123)	0.768^***^ (0.107)	Yes			20,019		0.000
		2017	−1.090^***^ (0.095)	0.779^***^ (0.109)	Yes			20,633		0.000
		2019	−1.309^***^ (0.169)	0.464^***^ (0.098)	Yes			19,873		0.000
	Imputed missing data	2013	−1.231^***^ (0.070)	0.749^***^ (0.077)	Yes			21,121	Yes	0.000
		2017	−1.110^***^ (0.064)	0.751^***^ (0.075)	Yes			21,623	Yes	0.000
		2019	−1.313^***^ (0.060)	0.456^***^ (0.070)	Yes			20,763	Yes	0.000
Heckman selection model	Without weights	2013	−0.686^***^ (0.035)	0.512^***^ (0.034)	Yes		Yes	26,511^g)^		0.000
		2017	−0.635^***^ (0.032)	0.483^***^ (0.033)	Yes		Yes	26,863^h)^		0.000
		2019	−0.729^***^ (0.041)	0.262^***^ (0.031)	Yes		Yes	26,423^i)^		0.000
	With weights	2013	−0.556^***^ (0.053)	0.380^***^ (0.049)	Yes		Yes	26,511		0.000
		2017	−0.570^***^ (0.061)	0.474^***^ (0.058)	Yes		Yes	26,863		0.000
		2019	−0.626^***^ (0.047)	0.210^***^ (0.056)	Yes		Yes	26,423		0.000
Logistic regression	Without weights	2013	−1.402^***^ (0.120)	1.121^***^ (0.171)	Yes	Yes^j)^		20,019		0.000
		2017	−1.265^***^ (0.105)	1.038^***^ (0.161)	Yes	Yes^j)^		20,633		0.000
		2019	−1.503^***^ (0.185)	0.550^***^ (0.141)	Yes	Yes^j)^		19,873		0.000
	With weights	2013	−1.138^***^ (0.114)	0.606^***^ (0.122)	Yes	Yes^k)^		20,019		0.000
		2017	−1.110^***^ (0.104)	0.802^***^ (0.133)	Yes	Yes^k)^		20,633		0.000
		2019	−1.171^***^ (0.095)	0.335^***^ (0.126)	Yes	Yes^k)^		19,075^l)^		
	Imputed missing data	2013	−1.398^***^ (0.121)	1.108^***^ (0.168)	Yes	Yes^j)^		21,121	Yes	0.000
		2017	−1.287^***^ (0.100)	1.010^***^ (0.154)	Yes	Yes^j)^		21,623	Yes	0.000
		2019	−1.515^***^ (0.175)	0.540^***^ (0.139)	Yes	Yes^j)^		20,763	Yes	0.000

*Significant at*

* *p < 0.1*,

** *p < 0.05*,

*** *p < 0.01; Standard errors reported in parentheses*.

a) *Values, norms, and beliefs (informal institutions) in asymmetry with formal rules (formal institutions)*;

b) *IPH, Informal Practices in the Health sector; Perception that informal practices (giving/taking bribes, abuse of power for personal gain) are widespread in the healthcare system*;

c) *Socio-demographic variables*;

d) *Variables related to healthcare users, included in the selection equation: age, education, household size*;

e) *Observations*;

f) *Multivariate imputations*;

g) *Censored: 6,492; Uncensored: 20,019*;

h) *Censored: 6,230; Uncensored: 20,633*;

i) *Censored: 6,550; Uncensored:19,873*;

j) *Cluster by country*;

k) *Country dummies*;

l) *Sweden excluded (no informal payments by patients reported in Sweden*).

*Source: own calculations based on data from Special Eurobarometer 397/Wave EB79.1 (2014, fieldwork 2013), Special Eurobarometer 470/Wave EB88.2 (2017, fieldwork 2017) and Special Eurobarometer 502/Wave EB92.4 (2020, fieldwork 2019)*.

To better display, the magnitude of the effect of the individual characteristics of patients on the likelihood to make informal payments over time, Figures A1–C1 provides the marginal effects of all the independent variables used in the analysis, while Figures A2–C2 shows the predicted probability of a “representative” patient in the EU-27 and UK to make informal payments in accordance with the value of the asymmetry index and view of patients on the widespread of the informal practices in the healthcare sector. According to Figures A1–C1, the misalignment between the formal and informal institutions has the largest effect on reducing the informal payments to healthcare practitioners in the public healthcare system. Indeed, in 2019, a low misalignment between patients' values/norms and formal rules decreases the probability of patients to make informal payments by approximately five percentage points. The perception on whether the informal practices are widespread in the public healthcare system has the second largest effect in 2013 and 2017, and slightly lower effect than the financial difficulties in 2019. As Figure C1 shows, perceiving that the informal practices are widespread in the public healthcare sector increases the probability to make informal payments by approximately two percentage points.

**Figure 1:**
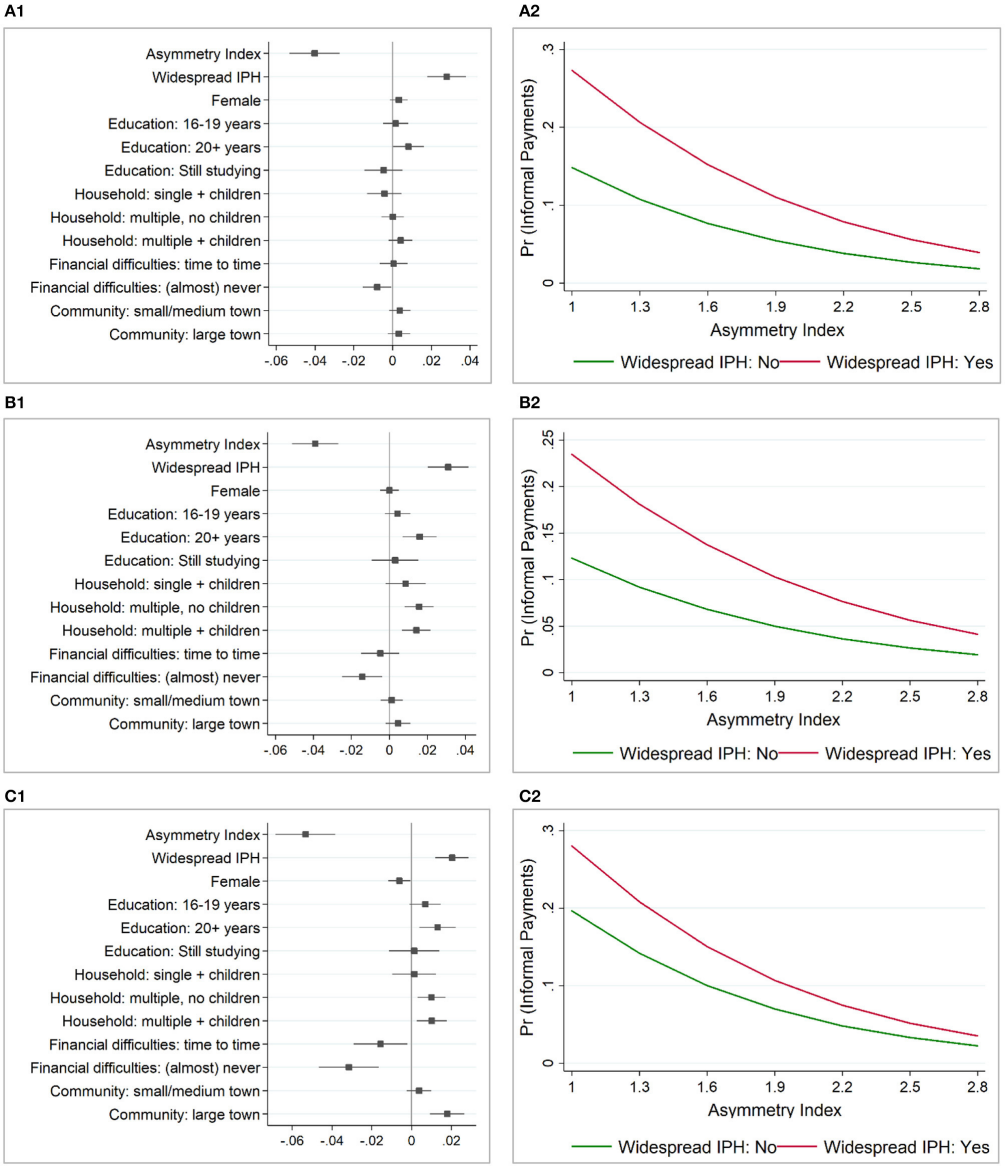
Marginal effects and predicted probability to make informal payments in the EU-27 and the UK. **(A1)** Marginal effects (2013). **(A2)** Predicted probability (2013). **(B1)** Marginal effects (2017). **(B2)** Predicted probability (2017). **(C1)** Marginal effects (2019). **(C2)** Predicted probability (2019). After multilevel logistic regression; Asymmetry Index–norms, values and beliefs (informal institutions) in asymmetry with formal rules (formal institutions); Widespread IPH (Informal Practices in the Healthcare sector)–perception that giving/taking bribes and the abuse of power for personal gain are widespread in the healthcare system; Predicted probability: fixed portion only—by a “representative” patient in EU-27 and UK (mean and mode of the socio-demographic variables: female, 16–19 years when stopped education, having (almost) never difficulties in paying bills and living in a multiple household with children in a small/middle sized town). Source: own calculations based on data from Special EB 397/Wave EB79.1 (2014, fieldwork 2013), Special EB 470/Wave EB88.2 (2017, fieldwork 2017) and Special EB 502/Wave EB92.4 (2020, fieldwork 2019).

Figures A2–C2 shows the predicted probability to make informal payments by these two most salient factors, namely: the asymmetry index and the perception on whether or not informal practices are widespread in the public healthcare sector. Similar results are observed over the analyzed period. When patients have a high misalignment (i.e., they are tolerant to corrupt behavior and find acceptable to give gifts, money, and to do favors to get a “free” public service) the probability of making informal payments in the healthcare is much higher than when they have a low misalignment. However, the social norms or the perception on the spread of informal practices in the healthcare sector also plays a pivotal role in predicting informal payments made by patients to healthcare practitioners in the public healthcare system. The effect is higher when the asymmetry is high, for the highest misalignment level (i.e., asymmetry index value of 1 or finding the corrupt behavior always acceptable) the predicted probability of making informal payments being one third higher for those who perceive that the informal practices are widespread in the healthcare system as compared to those who do not have the same perception. The effect is lower when the asymmetry is low (i.e., index value of 2.8), with a small difference in the predicted probabilities of making informal payments between those who perceive that informal practices are widespread in the healthcare sector and those who do not have the same perception.

## Discussion

This paper has advanced the institutional theory toward informal payments in healthcare by evaluating the role of social norms in the propensity of making such payments. In addition, the paper investigated the trends over time in the prevalence of informal payments made by patients as well as the trends in the effect of values, norms, and beliefs on these payments. The results show that, amongst the European member states and the UK, the prevalence of informal payments to healthcare practitioners in 2019 remained the same as in 2013, with 5% of the patients declaring that they have made such a payment. Furthermore, in most of the countries, there is an increase in the percentage of patients making informal payments, and only in three countries, a descending trend is observed. This calls for more effective policy measures aimed at reducing this practice.

Drawing from institutional theory, both the role of the formal–informal institutions misalignment and the role of social norms in reducing informal payments were explored in this paper. The findings underlined that, indeed both, the formal–informal institutions misalignment—as well as the social norms (measured here using the perception on whether the informal practices in the public healthcare sector are widespread in the society or not) play a pivotal role in explaining the likelihood of informal payments occurrence. The results are robust across the three analyzed survey waves. Therefore, to tackle informal payments made by patients to practitioners in the public healthcare sector it is required to reduce the misalignment between the patients' values, norms, and beliefs and the formal rules and to change the social norms. This involves changes in both informal institutions and formal institutions in order to close the gap between them. As such, improvements of formal institutions could focus on issues identified in previous studies such as underfunding, lack of wider economic and social development (16–18, 25). Indeed, previous studies identified a direct link between the prevalence of informal payments and low health expenditure, low range and accessibility of healthcare services, low health outcomes, and low quality of government (25, 26). Meanwhile, as the results of this study show, the role of informal institutions should not be neglected or underestimated. Both, individual and social norms need to be altered. In order to achieve this, awareness and information campaigns could be used to inform the patients of the risks involved in making this type of payments, on the fact that such payments are not required for receiving a proper treatment, or to advertise that other patients and the medical staff behave in a compliant manner. These campaigns could be targeted at the socio-demographic categories identified in this study (i.e., those with financial difficulties and those who are better educated). Also, another measure that can contribute to altering the values, norms, and beliefs of patients is represented by the use of normative appeals aimed at reducing the tendency of patients and practitioners in the public healthcare sector to pay or receive such payments. Furthermore, tax education to inform the citizens that an ethical behavior in paying their taxes would benefit the public services, healthcare included, by providing better salaries and better equipment. If successful, then the patients and the practitioners in the public healthcare services would no longer feel necessary this type of payments. Nevertheless, this paper has limitations. Firstly, no longitudinal data were available to allow causality inferences. Secondly, the share of informal payments by patients might be underestimated. Indeed, some respondents might not answer honestly when asked about this practice. Thirdly, in some countries (i.e., Cyprus, Luxembourg, and Malta), there are less number of respondents (about 500) and, therefore, the results should be cautiously interpreted. Fourth and finally, other potential drivers of informal payments (e.g., cultural determinants, motives for making/accepting informal payments, initiator of this type of payment) were not available to be tested. As such, future research is required to explore why patients decide to make such payments and why healthcare professionals accept these payments.

## Data Availability Statement

Publicly available datasets were analyzed in this study. This data can be found at: European Commission, Brussels (2016): Eurobarometer 79.1 (2013). TNS Opinion, Brussels [producer]. GESIS Data Archive, Cologne. ZA5687 Data file Version 3.0.0, https://doi.org/10.4232/1.12448; European Commission, Brussels (2018): Eurobarometer 88.2 (2017). TNS opinion, Brussels [producer]. GESIS Data Archive, Cologne. ZA6927 Data file Version 1.0.0, https://doi.org/10.4232/1.13005; European Commission, Brussels (2020): Eurobarometer 92.4 (2019). Kantar Public [producer]. GESIS Data Archive, Cologne. ZA7602 Data file Version 1.0.0, https://doi.org/10.4232/1.13652.

## Author Contributions

The author confirms being the sole contributor of this work and has approved it for publication.

## Funding

This work was supported by a grant of the Ministry of Research, Innovation and Digitization, CNCS/CCCDI—UEFISCDI, project number PN-III-P1-1.1-TE-2019-0163, within PNCDI III.

## Conflict of Interest

The author declares that the research was conducted in the absence of any commercial or financial relationships that could be construed as a potential conflict of interest.

## Publisher's Note

All claims expressed in this article are solely those of the authors and do not necessarily represent those of their affiliated organizations, or those of the publisher, the editors and the reviewers. Any product that may be evaluated in this article, or claim that may be made by its manufacturer, is not guaranteed or endorsed by the publisher.

## References

[1] Chereches R Ungureanu MI Sandu P Rus IA. Defining informal payments in healthcare: a systematic review. Health Policy. (2013) 110:105–14. 10.1016/j.healthpol.2013.01.010 23410757

[2] Stepurko T Pavlova M Gryga I Groot W. Making patients pay: informal patient payments in Central and Eastern European Countries. Front Public Health. (2015) 3:192. 10.3389/fpubh.2015.00192 26301214 PMC4528095

[3] Delcheva E Balabanova D McKee M. Under-the-counter payments for health care: evidence from Bulgaria. Health Policy. (1997) 42:89–100. 10.1016/S0168-8510(97)00061-4 10175625

[4] Ensor T. Informal payments for health care in transition economies. Soc Sci Med. (2004) 58: 237–46. 10.1016/S0277-9536(03)00007-8 14604610

[5] Mossialos E Dixon A Figueras J Kutzin J. Policy Brief-Funding Health Care: Options for Europe. Brussels: European Observatory on Health Care Systems (2002).

[6] Amiri MM Bahadori M Ravangard R. Factors affecting informal patient payments: a systematic literature review. Int J Health Gov. (2019) 24:117–32. 10.1108/IJHG-01-2019-0006

[7] Tomini S Groot W Pavlova M. Paying informally in the Albanian health care sector: a two-tiered stochastic frontier model. Eur J Health Econ. (2012) 13:777–88. 10.1007/s10198-011-0331-1 21691842 PMC3482461

[8] Lindkvist I. Informal payments and health worker effort: a quantitative study from Tanzania. Health Econ. (2013) 22:1250–71. 10.1002/hec.2881 23188621

[9] Khodamoradi A Ghaffari MP Daryabeygi-Khotbehsara R Sajadi HS Majdzadeh R, A systematic review of empirical studies on methodology and burden of informal patient payments in health systems. Int J Health Plann Manage. (2018) 33:e26–37. 10.1002/hpm.2464 29076562

[10] Horodnic AV Mazilu S Oprea L. Drivers behind widespread informal payments in the Romanian public health care system: from tolerance to corruption to socio-economic and spatial patterns. Int J Health Plann Manage. (2018) 33:e597–611. 10.1002/hpm.2509 29542181

[11] Stepurko T Pavlova M Gryga I Groot W. Informal payments for health care services—corruption or gratitude? a study on public attitudes, perceptions and opinions in six Central and Eastern European countries. Commu Post Commun Stud. (2013) 46:419–31. 10.1016/j.postcomstud.2013.08.004

[12] Baji P Pavlova M Gulácsi L Groot W. Does the implementation of official user charges help to eradicate informal payments—lessons to be learnt from the Hungarian experience. Front Public Health. (2015) 3:181. 10.3389/fpubh.2015.00181 26236705 PMC4505068

[13] Tomini SM Groot W Pavlova M Tomini F. Paying out-of-pocket and informally for health care in Albania: the impoverishing effect on households. Front Public Health. (2015) 3:207. 10.3389/fpubh.2015.00207 26380252 PMC4551817

[14] World Health Organization (WHO). WHO/Europe Assists Greece in Addressing Informal Payments in the Health Sector. (2017). Available online at: http://www.euro.who.int/ (accessed August 25, 2021).

[15] Pourtaleb A Jafari M Seyedin H Behbahani AA. New insight into the informal patients' payments on the evidence of literature: a systematic review study. BMC Health Serv Res. (2020) 20:14. 10.1186/s12913-019-4647-3 31902368 PMC6943960

[16] Cohen N. Informal payments for health care–the phenomenon and its context. Health Econ Policy Law. (2012) 7:285–308. 10.1017/S1744133111000089 21554778

[17] Gaal P McKee M. Fee-for-service or donation? Hungarian perspectives on informal payment for health care. Soc Sci Med. (2005) 60:1445–57. 10.1016/j.socscimed.2004.08.009 15652678

[18] Tambor M Pavlova M Golinowska S Sowada C Groot W. The formal-informal patient payment mix in European countries. Governance, economics, culture or all of these? Health Policy. (2013) 113:284–95. 10.1016/j.healthpol.2013.09.011 24149101

[19] Liaropoulos L Siskou O Kaitelidou D Theodorou M Katostaras T. Informal payments in public hospitals in Greece. Health Policy. (2008) 87:72–81. 10.1016/j.healthpol.2007.12.005 18249459

[20] Atanasova E Pavlova M Groot W. Out-of-pocket patient payments for public health care services in Bulgaria. Front Public Health. (2015) 3:175. 10.3389/fpubh.2015.00175 26217655 PMC4495311

[21] Vafaei Najar A Ebrahimipour H Pourtaleb A Esmaily H Jafari M Nejatzadegan Z. At first glance, informal payments experience on track: why accept or refuse? Patients' perceive in cardiac surgery department of public hospitals, northeast of Iran 2013. BMC Health Serv Res. (2017) 17:205. 10.1186/s12913-017-2108-4 28292289 PMC5351263

[22] Tahiri Z Toçi E Rrumbullaku L Hoti K Roshi E Burazeri G. Patients' evaluation of primary health care services in Gjilan region, Kosovo. J Public Health (Oxf). (2014) 36:161–9. 10.1093/pubmed/fdt041 23596194

[23] Stringhini S Thomas S Bidwell P Mtui T Mwisongo A. Understanding informal payments in health care: motivation of health workers in Tanzania. Hum Resour Health. (2009) 7:53. 10.1186/1478-4491-7-53 19566926 PMC2711965

[24] García PJ. Corruption in global health: the open secret. Lancet. (2019) 394:2119–24. 10.1016/S0140-6736(19)32527-9 31785827

[25] Williams CC Horodnic AV. Rethinking informal payments by patients in Europe: an institutional approach. Health Policy. (2017) 121:1053–62. 10.1016/j.healthpol.2017.08.007 28867153

[26] Horodnic AV Williams CC. Informal payments in the health services sector: prevalence and determinants. Serv Ind J. (2018) 38:841–55. 10.1080/02642069.2018.1450870

[27] Horodnic IA Williams CC. Tackling undeclared work in the European Union: beyond the rational economic actor approach. Policy Stud. (2019). 10.1080/01442872.2019.1649384

[28] Horodnic IA Williams CC. Evaluating policy approaches for tackling informal entrepreneurship. J Small Bus Enterp Dev. (2018) 26:595–611. 10.1108/JSBED-08-2018-0252

[29] Williams CC Horodnic IA. Trends in the Undeclared Economy and Policy Approaches. Brussels: European Commission (2020).

[30] Williams CC Öz-Yalaman G. Re-theorising participation in undeclared work in the European Union: lessons from a 2019 Eurobarometer survey. Eur Soc. (2021) 23:403–27. 10.1080/14616696.2021.1887915

[31] Horodnic IA. Tax morale and institutional theory: a systematic review. Int J Sociol Soc Policy. (2018) 38:868–86. 10.1108/IJSSP-03-2018-0039

[32] Ajzen I. The theory of planned behavior. Organ Behav Hum Decis Process. (1991) 50:179–211. 10.1016/0749-5978(91)90020-T

[33] European Commission. Special Eurobarometer 397—Corruption (Report). Brussels: European Commission (2014).

[34] European Commission. Special Eurobarometer 470—Corruption (Report). Brussels: European Commission (2017).

[35] European Commission. Special Eurobarometer 502—Corruption (Report). Brussels: European Commission (2020).

[36] Williams CC Horodnic AV. Evaluating the prevalence of informal payments for health services in Southeast Europe: an institutional approach. Southeast Eur Black Sea Stud. (2018) 18:345–65. 10.1080/14683857.2018.1487138

[37] Williams CC Horodnic AV. Explaining informal payments for health services in Central and Eastern Europe: an institutional asymmetry perspective. Postcommunist Econ. (2018) 30:440–58. 10.1080/14631377.2018.1442051

[38] Williams CC Horodnic IA. Extent and distribution of unregistered employment in the service industries in Europe. Serv Ind J. (2018) 38:856–74. 10.1080/02642069.2018.1481209

[39] Horodnic IA Williams CC. Evaluating the working conditions of the dependent self-employed. Int J Entrep Behav Res. (2019) 26:326–48. 10.1108/IJEBR-07-2018-0445

[40] Williams CC Horodnic IA. Evaluating the illegal employer practice of under-reporting employees' salaries. Br J Ind Relat. (2017) 55:83–111. 10.1111/bjir.12179

[41] Timofeyev Y Jakovljevic M. Fraudster's and victims' profiles and loss predictors' hierarchy in the mental healthcare industry in the US. J Med Econ. (2020) 23:1111–22. 10.1080/13696998.2020.1801454 32713224

[42] Stepurko T Pavlova M Gryga I Gaál P Groot W. Patterns of informal patient payments in Bulgaria, Hungary and Ukraine: a comparison across countries, years and type of services. Health Policy Plan. (2017) 32:453–66. 10.1093/heapol/czw147 27993960

[43] Stepurko T Pavlova M Gryga I Groot W. To pay or not to pay? a multicountry study on informal payments for health-care services and consumers' perceptions. Health Expect. (2015) 18:2978–93. 10.1111/hex.12281 25292329 PMC5810640

[44] Williams CC Horodnic IA Horodnic AV. Who is making informal payments for public healthcare in East-Central Europe? an evaluation of socio-economic and spatial variations. Eastern J Eur Stud. (2016) 7:49–61.

[45] Horodnic AV Ciobanu CI Williams CC Rodgers P. Assessing the Frequency of Informal Payments for Health Services in Lithuania. In: Horodnic IA Rodgers P Williams CC Momtazian L, editors. The Informal Economy: Exploring Drivers and Practices. London: Routledge (2018). p. 73–90.

[46] Solon G Haider SJ Wooldridge J. What are we weighting for? J Hum Resour. (2015) 50:301–16. 10.3368/jhr.50.2.301

[47] Winship C Radbill L. Sampling weights and regression analysis. Sociol Methods Res. (1994) 23:230–57. 10.1177/0049124194023002004

[48] Zyaambo C Siziya S Fylkesnes K. Health status and socio-economic factors associated with health facility utilization in rural and urban areas in Zambia. BMC Health Serv Res. (2012) 12:389. 10.1186/1472-6963-12-389 23145945 PMC3536624

[49] Morrissey K Kinderman P Pontin E Tai S Schwannauer M. Web based health surveys: using a Two Step Heckman model to examine their potential for population health analysis. Soc Sci Med. (2016) 163:45–53. 10.1016/j.socscimed.2016.06.053 27394193

