# Supplementary material for: Trends in Informal Payments by Patients in Europe: A Public Health Policy Approach
Source: Front Public Health. 2021 Nov 22;9:780337. doi: 10.3389/fpubh.2021.780337 (PMC8645776; doi:10.3389/fpubh.2021.780337)
Supplement: Supplementary file 1 [file Table_1.DOCX]

Supplementary Material

**Table S1.** Variables used in the analysis

| Variable | Definition | Variable type | | Mode or mean | | | Min, Max |
| --- | --- | --- | --- | --- | --- | --- | --- |
|  |  |  |  | *Special Eurobarometer* | | |  |
|  |  |  |  | 2013 | 2017 | 2019 |  |
|  |  |  |  | (N=21,121) | (N=21,623) | (N=20,763) |  |
| Informal payments in healthcare | Patients using informal payments for healthcare services | Dummy | - Informal payments: No - Informal payments: Yes | No: 95% | No: 95% | No: 95% | 0,1 |
| Asymmetry Index | Constructed index of the asymmetry between patient`s norms, values and beliefs (informal institutions) and formal rules (formal institutions) | Numeric |  | 2.76 | 2.79 | 2.77 | 1,3 |
| Widespread IPH | Patient`s perception on whether informal practices are widespread in the healthcare sector | Dummy | - Widespread: No - Widespread: Yes | No: 67% | No: 69% | No: 74% | 0,1 |
| Gender | Gender of the patient | Dummy | - Male - Female | Female: 55% | Female: 54% | Female: 55% | 0,1 |
| Age education ended | Age when the patient stopped full time education | Categorical | - Up to 15 years - 16-19 years - 20+ years - Still studying | 16-19 years: 43% | 16-19 years: 42% | 16-19 years: 41% | 1,4 |
| Household | Patient`s household (hh) composition, including marital status | Categorical | - Single hh without children - Single hh with children - Multiple hh without children - Multiple hh with children | Multiple hh with children: 34% | Multiple hh with children: 32% | Multiple hh with children: 31% | 1,4 |
| Financial difficulties | Difficulties faced by patients in paying household bills | Categorical | - Most of the time - From time to time - Almost never/never | Almost never/never: 61% | Almost never/never: 68% | Almost never/never: 72% | 1,3 |
| Community size | Patient`s area of residence | Categorical | - Rural area or village - Small or middle sized town - Large town | Small or middle sized town: 41% | Small or middle sized town: 44% | Small or middle sized town: 45% | 1,3 |

*Source*: own calculations based on data from Special Eurobarometer 397 / Wave EB79.1 (2014, fieldwork 2013), Special Eurobarometer 470 / Wave EB88.2 (2017, fieldwork 2017) and Special Eurobarometer 502 / Wave EB92.4 (2020, fieldwork 2019)

**Table S2.** Missing values - imputations

| Variable |  | *Special Eurobarometer*  2013 | | |  | *Special Eurobarometer*  *2017* | | |  | *Special Eurobarometer*  *2019* | | |
| --- | --- | --- | --- | --- | --- | --- | --- | --- | --- | --- | --- | --- |
|  |  | Complete | Imputed | Total |  | Complete | Imputed | Total |  | Complete | Imputed | Total |
| Informal payments in healthcare |  | 20,689 | 74 | 20,763 |  | 21,508 | 115 | 21,623 |  | 20,936 | 185 | 21,121 |
| Asymmetry Index |  | 20,530 | 233 | 20,763 |  | 21,384 | 239 | 21,623 |  | 20,986 | 135 | 21,121 |
| Widespread IPH |  | 20,763 | 0 | 20,763 |  | 21,623 | 0 | 21,623 |  | 21,121 | 0 | 21,121 |
| Gender |  | 20,763 | 0 | 20,763 |  | 21,623 | 0 | 21,623 |  | 21,121 | 0 | 21,121 |
| Age education ended |  | 20,465 | 298 | 20,763 |  | 21,343 | 280 | 21,623 |  | 20,836 | 285 | 21,121 |
| Household |  | 20,632 | 131 | 20,763 |  | 21,481 | 142 | 21,623 |  | 20,850 | 271 | 21,121 |
| Financial difficulties |  | 20,543 | 220 | 20,763 |  | 21,323 | 300 | 21,623 |  | 20,821 | 300 | 21,121 |
| Community size |  | 20,760 | 3 | 20,763 |  | 21,612 | 11 | 21,623 |  | 21,102 | 19 | 21,121 |

*Source*: own calculations based on data from Special Eurobarometer 397 / Wave EB79.1 (2014, fieldwork 2013), Special Eurobarometer 470 / Wave EB88.2 (2017, fieldwork 2017) and Special Eurobarometer 502 / Wave EB92.4 (2020, fieldwork 2019)
